# Integrated Approach to Evaluate the Association between Exposure to Pesticides and Idiopathic Premature Thelarche in Girls: The PEACH Project

**DOI:** 10.3390/ijms21093282

**Published:** 2020-05-06

**Authors:** Lucia Coppola, Sabrina Tait, Lorella Ciferri, Gianluca Frustagli, Carmine Merola, Monia Perugini, Enrica Fabbrizi, Cinzia La Rocca

**Affiliations:** 1 Center for Gender-Specific Medicine, Italian National Institute of Health, 00161 Rome, Italy; lucia.coppola@guest.iss.it (L.C.); sabrina.tait@iss.it (S.T.); 2 Department of Physiology and Pharmacology V. Erspamer, Sapienza University of Rome, 00185 Rome, Italy; 3 ASUR MARCHE Area Vasta 4, 63822 Porto San Giorgio (FM), Italy; l.ciferri@alice.it; 4 Core Facilities Service, Italian National Institute of Health, 00161 Rome, Italy; gianluca.frustagli@iss.it; 5 Faculty of Bioscience and Technology for Food, Agriculture and Environment, University of Teramo, 64100 Teramo, Italy; cmerola@unite.it (C.M.); mperugini@unite.it (M.P.); 6 Pediatric Unit, Maternal Infant Department “A. Murri” Hospital, ASUR MARCHE Area Vasta 4, 63900 Fermo, Italy; enrica.fabbrizi@libero.it

**Keywords:** endocrine disruptors, pesticides, dietary exposure, idiopathic premature thelarche

## Abstract

Several pesticides are recognized as endocrine-disrupting chemicals (EDCs) since they can interfere with the dysregulation of sexual, thyroid and neuro-endocrine hormones. Children are particularly vulnerable to the adverse effects of EDCs due to their developmental stage, peculiar lifestyle and dietary habits. In this context, the exposure to pesticides represents an important risk factor associated with early development. This study deals with the possible association between exposure to pesticides and idiopathic premature thelarche in girls from areas of intensive agriculture practice in the Centre of Italy. An integrated approach was set up, including: (i) a case-control study on girls with idiopathic premature thelarche; (ii) the evaluation of multiple pesticides exposure in girls; (iii) the evaluation of multiple pesticides in food; (iv) the dietary intake of pesticide residues; (v) the assessment of toxicological effects of widely used pesticides by in vitro model. Data integration will provide an estimate of the predictive risk of potential effects on girls’ health, linked to dietary intake.

## 1. Introduction

In agriculture, pesticides, including acaricides, insecticides, herbicides and fungicides, are used to control insects, weeds, fungi and rodents that can damage crops. Despite the beneficial effects on plant production, pesticides can represent a risk for non-target organisms, including humans [1].

By a systematic evaluation of epidemiological studies concerning the association between pesticide exposure and health outcome, the European Food Safety Authority (EFSA) highlighted that human exposure to pesticides is linked to a large number of health implications, including cancer, neurological diseases and mental development, respiratory and cardiovascular diseases, diabetes reproductive diseases and endocrine disorders. In particular, the latter deserve special attention and further studies [2].

Reproductive adverse effects of pesticides are related to sex. In men, pesticides exposure has been associated to reduced testosterone synthesis and sperm quality and motility, as well as testicular cancer; in women, the main effects were observed on the alteration of ovarian cycle, the imbalance of hormone concentrations and fertility, and the induction of spontaneous abortion [3,4].

However, the main concern is for children, who are particularly vulnerable to the adverse effects of pesticides with endocrine-disrupting activities, due to their peculiar lifestyle, dietary habits and developmental stage, such as in puberty.

### 1.1. Pesticides and Pubertal Development

Besides genetic and physiological factors, environmental factors can also contribute to the dysregulation of puberty onset at central level, on hypothalamus, or at the peripheral level on breast and gonads. In the last decades, a progressive shortening of the time of puberty in girls and a consequent increased incidence of precocious puberty (i.e., the development of pubertal changes before the age of 8 years) and premature thelarche (i.e., early breast development before the age of 8 years) were observed worldwide [5]. Since the onset of puberty is regulated by the activation of hormones and neurotransmitters network of hypothalamus-pituitary-gonadal axis (HPG axis), any imbalance perturbing this phase may detrimentally affect sexual development. The release of the gonadotropin-releasing hormone (GnRH) by the hypothalamus, induces the secretion of the gonadotropins luteinizing hormone (LH) and follicle-stimulating hormone (FSH) from the anterior pituitary gland, that in turn regulate the hormonal production by the gonads [6]. In girls, the increase in gonadotropins stimulates ovaries to secrete androgens from theca follicle cells and estradiol (E2) from granulosa cells, which then promotes breast development [7].

Several pesticides can act as endocrine-disrupting chemicals (EDCs), displaying agonistic and anti-agonistic activities toward both estrogen receptors (ERα and ERβ) and androgen receptor (AR) (i.e., chlorpyrifos methyl, propiconazole, carbaryl, methiocarb), as well as interaction with aromatase functions and steroid hormone metabolism (i.e., cyproconazole, prochloraz) [8]. A transactivation ER assay using a human breast cancer cell line (MVLN) showed the estrogenic activities of terbuthylazine, propiconazole, prothioconazole, cypermethrin and malathion while also demonstrating that bitertanol, propiconazole and mancozeb have anti-androgenic activity and terbuthylazine, propiconazole and prothioconazole can act as aromatase activity inducers [9,10]. Also relevant is the interaction with the pregnane X receptor that entails the implication of pesticides on cellular metabolism and the detoxification process by regulating the transcription of genes involved in the microsomal cytochrome P450 and conjugation enzymes (i.e., fenbuconazole, tebuconazole), thus possibly affecting steroid hormones homeostasis. The thyroid homeostasis and signaling are also affected by mancozeb, zineb, dimethoate, trichlorfon and malathion with adverse impact on hypothalamus-pituitary-thyroid axis [11].

The impact of pesticides exposure on premature thelarche and precocious puberty, due to their endocrine disruptor characteristics, has been considered in some studies. Exposure of female rats to chlorpyrifos, increased LH, FSH and E2 serum levels, possibly due to an effect on anterior pituitary function [12]. Similarly, imidacloprid increased FSH and decreased LH and progesterone serum levels in treated female rats, probably due to an alteration in GnRH release, with consequences on ovary morphology [13]. In an in vitro study on swine granulosa cells, glyphosate decreased E2 and increased progesterone secretion [14].

Effects evident at puberty may have had an origin in utero since many pesticides may cross the placental barrier, as observed in girls whose mothers worked in greenhouses, displaying an early breast development with concomitant higher serum levels of androstenedione, a precursor of both androgens and estrogens, and lower levels of the anti-Mullerian hormone [15], which generally increases during the pre-pubertal period. In utero and lactational exposure to the anti-androgenic vinclozolin altered rat mammary gland development evident at puberty, with increased cell proliferation and excessive ductal branching [16].

Chlorpyrifos was demonstrated to affect the mammary gland in female adult rats by increasing cell proliferation and the number of ducts as well as progesterone receptor (PgR) expression involved in promoting ductal development [17]. A similar effect was also observed in vitro model using a human breast cancer cell line (MCF-7), by a mechanism possibly involving the phosphorylation of ERα [18].

### 1.2. Multiple Pesticide Residues Exposure

Several studies demonstrated that pesticide mixtures can elicit additive and synergic effects, e.g., the combination of organophosphorus and organochlorine pesticides increased the effect on steroid metabolism enzymes (CYP3A4 and CYP1A2) due to a dose additivity. Mixtures of pesticides also exerted additive estrogenic or anti-androgenic activity in vitro [9]. On the contrary, the interaction between triazine herbicides and prochloraz determined antagonistic effects on aromatase enzyme [19].

This evidence is highly relevant since the general population can be exposed to multiple pesticide residues, especially via food consumption. The 2017 monitoring data on pesticide residues in food indicated that about the 40% of analysed samples had residue levels at or below the maximum residue limit (MRL) tolerated in food and feed of plant and animal origin for several pesticides. However, data showed the presence of multiple residues in a single food commodity, due to the application of various pesticides against different pests [20,21].

The monitoring of pesticide residues in food by multiresidue analysis procedures is nowadays a primary objective in pesticide detection in order to extensively evaluate food quality and dietary human exposure [22]. Despite the difficulty to develop a multiresidue methods because of different polarity, solubility and volatility of compounds, in recent years, the QuEChERS extraction technique and mass spectrometry have made relevant progress in pesticide analysis being very accurate, rapid, sensitive and selective techniques for multiresidue determination [23].

As the multiple residue exposure may pose a risk to human health, data on dietary exposure, internal exposure levels and toxicological effects and interactions of pesticide mixtures can contribute to a more complete cumulative risk evaluation, according to EFSA criteria and methodology [24,25,26].

In this frame, the project “Integrated approach to evaluate children agricultural pesticide exposure and health outcome” (PEACH project, funded by the Italian Ministry of Health, RF-2016-02364628) aims to investigate the possible association between combined pesticide exposure and idiopathic premature thelarche (IPT) in girls and to explore molecular alterations implied in possible early human breast response to pesticides exposure by in vitro model.

With this respect, the approach of the project includes the following implementation actions: (a) the realization of a prospective case-control study on IPT in specific areas of central Italy characterized by intensive agriculture practices and by an increased incidence in the last 10 years of idiopathic premature thelarche in girls, as noticed by the paediatricians operating in these areas; (b) the analytical determination of a selected panel of pesticides and their metabolites in urine samples of enrolled girls, as well as a number of selected pesticides in food commodities locally produced and consumed by the enrolled girls; (c) the assessment of the dietary exposure of girls to the selected pesticides; (d) the evaluation of the potential adverse effects of widely used pesticides (i.e., chlorpyrifos, glyphosate and imidacloprid) by an in vitro model of the main target organ of disease, i.e., human breast cell line, at real exposure concentrations occurring in children.

The strength of the project is the ability to combine different approaches, namely case- control study, dietary exposure and in vitro studies, to provide scientific information to support risk assessment of multiple pesticides exposure in children, as a possible hazard linked to increasing cases of the dysregulation of puberty onset. The project started at the end of 2018 and it will be concluded at the end of 2021; at present, the activities within each action are ongoing. The present report aims to illustrate the approach set up by the project to integrate human and toxicological data, and to describe the state of art of the activities.

## 2. Methodology

### 2.1. Ethics Committee Approval

The project was evaluated by ethics committees as the main activity of the project concerns the collection of information and samples from girls. The ethics committees of the Istituto Superiore di Sanità (Italian National Health Institute), as project coordinator, and Marche Region, as the beneficiary in charge of the case-control study, approved the project and the informed consent (RF-2016-02364628, Prot N°PRE-4203, 17 April 2018; ASUR CERM 2018-190, respectively). The informed consent will be signed by the mothers of girls before the enrolment in the study and it also includes a section devoted to illustrating the study to girls through appropriated drawings. 

### 2.2. Geographical Area Selection and Agricultural Activities 

Specific areas in the Centre of Italy (Fermo and Macerata Districts, Marche Region) are considered in the study, both characterized by agriculture practice in farms and in private vegetable gardens.

Data on the type of cultivations in the Fermo and Macerata areas and the agrochemicals used on these cultivations were obtained by the local agronomists. Specifically, the main crops in the areas include cereals (e.g., wheat, barley, oats, corn, sorghum), sunflowers, leaf and cruciferous vegetables (e.g., cabbage, cauliflower, broccoli, radish, spinach, chicory, chard, lettuce), horticultural tomato, stone fruit (e.g., cherry; peach, plum, apricot), pome fruit (e.g., apples, pears, loquat). 

Among the several pesticides used in these types of cultivations we selected 44 compounds mainly used to be analysed in foods collected for this study (Table 1).

### 2.3. Case-Control Study

A case control study will be performed to measure pesticide levels in girls and to correlate pathology with individual and environmental factors (dietary habits, agricultural practices, related use of pesticides, land use).

The case-control study is conducted on girls with idiopathic premature thelarche matched for age with healthy girls as controls (*n* = 60 + 60), living in the selected areas (Fermo and Macerata Districts, Marche Region, Italy). Idiopathic premature thelarche girls are enrolled at the Fermo and Civitanova Marche Hospitals by the paediatric endocrinologist, as responsible of the case-control study within the project. During the medical examination of girls, the paediatric endocrinologist verifies the following conditions, including the differential diagnosis of true early puberty and premature thelarche, for the inclusion of patients in the study: age: 2–7 years, breast development (Tanner stage II), absence of other signs of puberty, normal stature, normal growth rate, age bone corresponding to chronological age or advanced of no more than 1 year, pelvic ultrasound consistent with pre-puberty (uterine longitudinal length and volume <3.5 cm and <1.8 mL), prepuberal Gonadotropin-releasing Hormone Stimulation Test (GnRH test, peak FSH value higher than LH, peak LH value <5 mIU/mL). The GnRH hormone test is performed by taking blood samples at 15th, 30th, 60th, and 120th minutes following intravenous administration of Relefact LH-RH (Sanofi-Aventis Deutschland GmbH, Frankfurt am Main, Germany.) at 100 µg/m2 diluted 1:10 with 0.9% physiological solution. In order to exclude other forms of precocious puberty, 17-β estradiol, alpha-fetoprotein, human chorionic gonadotropin, anti-Mullerian, thyroid and adrenal hormones are evaluated. All hormones are measured by the immune-chemiluminescence method according to manufacturer’s protocols (Beckman Coulter, Brea, CA 92821, USA), with detection limit (LOD) = 0.2 UI/mL and linearity in the range of 0.2–200 UI/mL. Moreover, 10 < BMI (Body Mass Index) > 75 and no concomitant therapy are applied as criteria for the enrolment of both cases and controls. The enrolment of healthy girls (controls) is carried out by the family pediatricians of the Italian National Health System in the same areas.

Each enrolled subject was asked to sign the consent, to collect a first morning urine sample and fill in the Food Frequency Questionnaire (FFQ) and the food diary during the day before the urine sampling.

The family pediatricians who joined the project, were trained by the responsible pediatric endocrinologist about the design of the study, inclusion/exclusion criteria to be applied for the enrolment of girls, the sampling of urine, and the management of documents and tools needed for the study, i.e., informed consent, FFQ, food diary and kit for urine sampling.

A few documents are provided to pediatricians to be submitted to the family of the enrolled girls, including:

1. An information sheet for the family of healthy controls (with the aim of encouraging participation), which describes the purpose of the study, the aim of urine sampling and the FFQ, and the confidentiality and management of personal data;
2. Informed consent for parents;
3. Information sheet for the child, like that presented to parents but with simple drawings and sentences and including informed consent;
4. Indications for the enlisted person on how to fill in the FFQ and the food diary as well as the instructions for collecting the urine samples;
5. The FFQ and the food diary.

The pediatricians were requested to select eligible candidates during their routine activity, presenting them the study and asking them to sign the informed consent. Moreover, they explain to girls’ mothers how to fill in the questionnaire/food diary, and how to collect first morning urine samples for the analytical determination of pesticide metabolites levels.

For each enrolled subject, an alphanumeric code was created to accomplish the ethics committee requirements concerning data protection and anonymity and assigned to urine sample and FFQ.

To date, 33 girls affected by premature thelarche and 22 age-matched healthy girls were enrolled and the related urine samples and FFQ were collected. The urine samples were frozen at −20 °C and then sent in batches to the laboratory for chemical analysis. The FFQ and food diary were sent to the project operative unit at Italian National Institute of Health for the elaboration data to plan the food sampling.

### 2.4. Food Frequency Questionnaire and Food Diary

A questionnaire was prepared in a paper form to collect both personal and residence area data and food consumption information.

Specifically, the questionnaire was structured in three parts. In the first part, personal information (e.g., age, body weight) and information on residential environment (e.g., proximity to cultivated fields, presence of private vegetable garden) are required. The second part is the FFQ. It includes one table for each food category considered in the official national and European control activities (i.e., vegetables, fruit, cereals, fish, meat, dairy products, eggs and honey) [27,28]. Food commodities listed within the categories were selected considering the information obtained by both the local agronomists about crops grown in the study area, and the paediatricians involved in the study, about the food items consumed by the children aged 2–7 years. Each food commodity has been associated to the alphanumeric code as reported by the European Food Safety Authority standardized system of food classification and description (FoodEx 2 EFSA database) to facilitate the comparison of data [29].

In the FFQ, each enrolled girl must specify the commodities consumed by indicating the frequency of consumption (daily, weekly, monthly), the usual portion consumed (small, medium, large) and the place of purchase/production (supermarket, farm or private garden).

The last part of the questionnaire is a food diary where detailed information on food consumed in the 24 h before the urine sampling must be indicated.

After the validation, the final version of the FFQ and food diary was sent to the local paediatricians involved in the girls’ enrolment.

The FFQs filled in by the enrolled girls were checked for data consistency; data entry was then performed in the online database, which was developed based on the paper version. The online database was created to properly assist the data entry and consistency check process. Due to the large number of information fields in a single questionnaire (much more than 1000), general-purpose application software (i.e., spreadsheets or similar) was deemed inadequate for this task. An ad hoc web application was developed, providing a suitable and user-friendly interface, especially designed to minimize input errors and having full editing capabilities. The application was developed in the well-known HTML-Javascript-PHP-MySQL-Apache framework, exclusively using open source software. Using the application, data are first stored into a relational database on a server and subsequently exported (via dedicated functions) into files in the Tab-Separated Values (TSV) format, suitable for further processing through scientific statistical analysis software.

To date, the sampling of summer vegetable and fruit consumed by the first 55 enrolled girls was completed. On the basis of data elaborated from their questionnaires, a consistent number of commodities were collected for both categories: chicory, chard, carrots, tomatoes, lettuce, zucchini, green beans, cucumbers, fennel, aubergines, potatoes, peppers, from 8 local farms and 10 private vegetable gardens; and peach, apricot, strawberries, cherries, loquat, from 7 local farms and 4 private vegetable gardens. The samples were sent under controlled temperature +4 °C to the laboratory for chemical analysis.

### 2.5. Food Sampling Plan and Dietary Exposure Assessment

Particular attention is given to the consumption of local foods, as a possible source of dietary exposure to pesticides in children living in the considered areas. Consequently, the defined food sampling plan foresees the purchase of food commodities at the same local farms or private vegetable gardens, as indicated by the enrolled girls; in addition, fruit and vegetables are purchased in summer and in winter, due to the different pesticides use during the year, as reported by the local agronomists.

Dietary exposure assessment of the enrolled girls to pesticides will be performed considering the type and quantity of food items consumed, as reported in the FFQ, the analytical levels determined by the project in locally produced foods and the exposure data from the conservative deterministic EFSA Pesticide Residues Intake Model (EFSA PRIMo) [30].

### 2.6. Chemical Analysis

The analyses for pesticide quantification (Table 1) are performed on all food samples coming from the sampling plan. Each sample is washed with ultrapure water before the homogenization. Commodities are divided in several groups following the SANTE/12682/2019 [31] document and each group is washed by ultrapure water and uniformly homogenized using an Osterizer. Samples are frozen (−20 °C) until analysis. The extraction is performed using the QuEChERS extraction technique and the supernatant is transferred to a clean tube and analyzed immediately by Liquid Chromatography-Tandem Mass Spectrometry (LC-MS/MS) or Gas Chromatography-Tandem Mass Spectrometry (GC-MS/MS).

For the urine, the creatinine is determined by colorimetric method Jaffé without deproteinization. The method is based on the reaction of creatinine, in an alkaline environment, with picrate to give a coloured compound whose intensity is proportional to the creatinine concentration in the sample. A spectrophotometer is used to quantify the creatinine values.

The determination of pesticides and their metabolites (except for the glyphosate and aminomethylphosphonic acid) in urine (Table 2) implies extraction by the solid phase extraction of the pesticides from the matrix. The final extract is injected in LC-MS/MS.

To determine the presence of glyphosate and aminomethylphosphonic acid (AMPA), a derivatization with 9-fluorenylmethyl chloroformate (FMOC-Cl) is applied and the final extracts injected in LC-MS/MS.

The analysis of both the panel of pesticides in food and the selected pesticide and their metabolites in urine samples is still in progress.

Creatinine levels were measured in the 55 urine samples collected until now; the results show the levels are within the normal range of 0.3–3 g/L [32].

### 2.7. Toxicological Study

Since the premature thelarche is characterized by an early breast development before the age of 8 years, the human cancer breast cell line, MCF7, was selected as the in vitro model representative of the target organ of the pathology. Consequently, MCF7 is used for the toxicological evaluation and the mode of action investigation of three pesticides, selected among the ones most used in agriculture practices in the area under study (see Section 2.2), namely chlorpyrifos, imidacloprid and glyphosate, alone or in mixture. 

To obtain comparable data with previous in vitro studies [18,33,34,35,36], cells are treated with the three pesticides as parent compounds, for 48 and 72 h, at 10-fold dilutions spanning real exposure concentrations occurring in children as derived by available epidemiological reports [37,38,39,40,41]. To prepare the mixtures of the three pesticides, we considered the blend of the mean detected level in children for each compound as a reference central concentration, and 10-fold dilutions were prepared accordingly. As a first step, cell vitality and cell proliferation were performed, respectively, by measuring the mitochondrial activity of living cells (MTS assay) and labelling the DNA within living cells (CyQuant assay) in three independent experiments (different cell passages) for each assay. The elaboration of the results, both as a dose–response curve fitting and a statistical evaluation of treated vs. control samples, is in progress.

Furthermore, the potential adverse effects of the three pesticides, alone or in mixture, will be evaluated treating the cells for 72 h in three independent experiments, at not cytotoxic concentrations, by the gene expression assessment of a panel of selected nuclear receptors (i.e., ERα, ERβ, AR, AhR and PgR) related to the disease under study, performed by real-time PCR (qPCR). Specific ELISA assays will be also performed to assess any hormonal imbalance (i.e., 17β-estradiol) due to pesticide treatment.

Any additional experiments not foreseen in the project, e.g., treatment with pesticide metabolites and/or the evaluation of further biomarkers of effect, will be considered when the experimental plan is completed.

### 2.8. Statistical Analysis Plan

Descriptive analyses and comparison between children groups (*p* < 0.05) will be carried out using the STATA 16.0 software (StataCorp) by parametric or non-parametric analysis according to data distribution. The relationship between idiopathic premature thelarche, pesticides internal levels, behavioral risk factors, environmental and food exposure to pesticides will be analysed by uni- and multivariate regression models including spatial correlates. Maps on land use and urbanization will be layered with girls’ geolocation and population density at municipality level data (ISTAT). Cases distribution, the relationship between cases and risk factors of the residence area will be explored by spatial statistics and clustering.

Dose–response curve fitting of in vitro toxicological assays will be performed by Prism 5.0 software (GraphPad Software Inc. San Diego CA USA). Evaluation of the statistical differences between treated and control cell samples will be performed by ANOVA with the post-hoc Dunnet’s test where appropriate, using STATA 16.0 software and setting significance at *p* < 0.05.

## 3. Expected Results

The main outcome of the PEACH project will be the development of a model to evaluate the association between girls’ exposure to pesticides and the onset of idiopathic premature thelarche based on an integrated approach. The integration of the scientific results will provide an estimate of the predictive risk of the potential effects of exposure to multiple pesticides on children’s health. Relevant results will be achieved:

1. The determination of urinary metabolite levels of selected pesticides in girls;
2. The determination of multiple pesticide levels in specific food groups from farms and private vegetable gardens;
3. Data on environmental characteristics and children’s food habits, as obtained from questionnaires;
4. Data on children pesticides dietary intake;
5. Toxicological evaluation of the endocrine effects of pesticides on the main target organ.

The developed model for the evaluation of exposure and public health impact will validate suitable tools to be considered in the risk assessment, easily transferable to similar environmental conditions as well as health warnings.

The project team, in collaboration with agronomists, will support campaigns of information for farmers on alternative uses or restrictive practices for the substitution of pesticides in agricultural production, but also for citizens toward the correct and prudent use of pesticides and alternative products or practice in private vegetable gardens. The overall action in promoting the right information will contribute to reducing children’s, workers’ and the general population’s exposure, thus protecting public health.

## Figures and Tables

**Table 1:** Pesticides analysed in food groups.

Chemical Class	Compounds	Chemical Class	Compounds
Dithiocarbamates	Mancozeb Metiram Penncozeb Propineb Thiram Zineb	Triazole	Cyproconazole Epoxiconazole Fenbuconazole Myclobutanil Penconazole Propiconazole Tebuconazole Thiabendazole Tricyclazole
Carbamates	Aldicarb Sulfone Aldicarb Sulfoxide Carbaryl Coumaphos Formetanate Isoprocarb Methiocarb Pirimicarb	Organophosphate	Chlorpyrifos-methyl Chlorpyrifosethyl Dimethoate Omethoate Phosmet Malathion Terbufos Terbufos sulfone Trichlorfon
Avermectine	Abamectin Emamectin	Neonicotinoid	Acetamipirid Imidacloprid Thiacloprid Thiamethoxam Clothianidin
Imidazole	Prochloraz	Phosphonoglycine	Glyphosate
Anilide	Boscalid	Thiophanate	Thiophanate-methyl
Benzodioxoles	Fludioxonil		

**Table 2:** Metabolite and parent pesticides analysed in urine samples.

Metabolite	Parent	Metabolite	Parent
Dimethylphosphate (DMP) Dimethylthiophosphate (DMTP) Phosmet oxon	Clorpyriphos-metile Phosmet	6-chloronicotinic acid (6CNA)	Acetamipirid Thiacloprid Imidacloprid
3-Chloro-4-methylumbelliferone (CMHC) Diethylphosphate (DEP) Diethylthiophosphate (DETP)	Coumaphos	Ethylene thiourea (ETU)	Mancozeb Metiram Penncozeb Thiram Zineb
Sum of B1a, B1b E B1A 8,9Z	Abamectin	2,4,6-Trichlorophenol Aminomethylphosphonic (AMPA)	Glyphosate
2,4,6-Trichlorophenol	Prochloraz	Malaoxon Malathion dicarboxylic acid (MDA)	Malathion
Propylenethiourea (PTU)	Propineb	Methiocarb sulfone e sulfoxide	Methiocarb
5-Hydroxythiabendazole	Thiabendazole	Pirimicarb-desmethyl	Pirimicarb
3,5,6-Trichloro-2-pyridinol (TCP) Diethylphosphate (DEP) Diethylthiophosphate (DETP)	Clorpyriphos-ethil Terbufos		

## Abbreviations

AR	Androgen Receptor
BMI	Body Mass Index
E2	Estradiol
EDC	Endocrine Disrupting Chemical
EFSA	European Food Safety Authority
ER	Estrogen Receptor
FFQ	Food Frequency Questionnaire
FSH	Follicle-stimulating hormone
GnRH	Gonadotropin-releasing Hormone
HPG axis	Hypothalamus-pituitary-gonadal axis
IPT	Idiopathic Premature Thelarche
LH	Luteinizing hormone
MRL	Maximum Residue Level
PgR	Progesterone Receptor
PRIMo	Pesticide Residues Intake Model
